# P02.29. Laser Doppler perfusion imaging to test effects of Lifewave energy enhancer patch on microvascular regulation

**DOI:** 10.1186/1472-6882-12-S1-P85

**Published:** 2012-06-12

**Authors:** A Baldwin

**Affiliations:** 1 University of Arizona, Tucson, USA

## Purpose

LifeWave Corp has developed non-transdermal energy enhancer patches that stimulate acupuncture points in order to heighten energy by enhancing the autonomic regulation of blood vessels and hence improving circulation. A laser Doppler perfusion imager was used to determine microvascular perfusion of the index, middle and ring finger tips pre and post application of the patches on wrist acupuncture points TB5 and P6. It was hypothesized that application of the patches would significantly alter perfusion in fingertips.

## Methods

Twenty healthy individuals (5 male and 15 female, 30-69 years of age) were recruited to participate in this randomized double blind study. Ten subjects received active patches and 10, placebo patches. Both hands of each subject were scanned before application of the patches (baseline), immediately after, and at l0 minutes, 4 hours and 8 hours after application at an ambient temperature of 24°C. The process was repeated the next day using new patches. All images were digitized and averaged over the 3 fingertips of each hand for each session. Repeated measures analysis of variance and pair-wise multiple comparisons were used to determine significant temporal differences in average perfusion.

## Results

On day 1, subjects in the active group showed no changes in perfusion compared to baseline whereas control subjects showed a significant increase (p<0.05) in left hand perfusion 10 min after patch application. On day 2, subjects in the active group showed a significant increase in left hand perfusion 4 hours after patch application whereas control subjects showed no change throughout the day.

## Conclusion

Placement of patches, per se, may cause a temporary relaxation of subcutaneous blood vessels. Placement of active patches causes a small, delayed microvascular relaxation in healthy subjects at room temperature. Experiments performed at different temperatures may produce more definitive results.

